# COMPARISON OF AGE AND BIOLOGICAL SEX MORTALITY TRENDS BETWEEN ADULTS WITH AND WITHOUT DOWN SYNDROME

**DOI:** 10.1093/geroni/igz038.1276

**Published:** 2019-11-08

**Authors:** James D Stevens, James D Stevens, Scott D Landes, Margaret A Turk

**Affiliations:** 1 Syracuse University Department of Sociology, Syracuse, New York, United States; 2 Syracuse University, Syracuse, New York, United States; 3 SUNY Upstate, Syracuse, New York, United States

## Abstract

Distinct mortality trends emerge from comparisons of mean and median age at death and specific causes of death between adults with and without cerebral palsy. We compare standardized mortality odds ratios (SMORs) for 20 leading causes of death for 11,895 adults with cerebral palsy and 13,047,988 without cerebral palsy in the US between 2012 and 2016. Male and female decadents with cerebral palsy died significantly younger than male and female decadents without cerebral palsy, and were more likely to die from respiratory diseases, choking, and unknown causes. Public health and preventive care efforts should account for respiratory, swallowing, and nutrition risks, as well as mortality trends’ variation across age and biological sex. The CDC and WHO could better surveil this population’s health and mortality by disallowing certifiers from using cerebral palsy as the underlying cause of death as the practice leads to high rates of unknown causes of death.

